# Breeding‐Related Changes in Social Interactions Among Female Vulturine Guineafowl

**DOI:** 10.1002/ece3.70943

**Published:** 2025-01-31

**Authors:** Tobit Dehnen, Brendah Nyaguthii, Wismer Cherono, Neeltje J. Boogert, Damien R. Farine

**Affiliations:** ^1^ Centre for Ecology and Conservation University of Exeter Penryn UK; ^2^ Department of Collective Behavior Max Planck Institute of Animal Behavior Konstanz Germany; ^3^ Department of Ornithology National Museums of Kenya Nairobi Kenya; ^4^ Mpala Research Centre Nanyuki Kenya; ^5^ Department of Wildlife, School of Natural Resource Management University of Eldoret Eldoret Kenya; ^6^ Department of Evolutionary Biology and Environmental Studies University of Zurich Zürich Switzerland; ^7^ Division of Ecology and Evolution, Research School of Biology Australian National University Canberra Australian Capital Territory Australia

**Keywords:** dominance, reproduction, social interactions, social transitions, temporal dynamics

## Abstract

Agonistic and affiliative interactions with group members dictate individual access to resources, and investment in competing for resources is often traded off with other needs. For example, reproductive investment can reduce body condition and, thereby, an individual's ability to win future agonistic interactions. However, group members may also alter their behaviour towards reproductive individuals, such as becoming more or less aggressive. Here, we investigated the social consequences of reproduction in female vulturine guineafowl *Acryllium vulturinum* , a plural breeder in which females disperse and are subordinate to males. We found opposing patterns for within‐ and between‐sex dominance interactions experienced by females from before to after breeding. Specifically, breeding females became far less likely to win dominance interactions with non‐breeding females after breeding than before breeding, but received considerably fewer male aggressions than non‐breeding females after breeding. Despite a limited sample size, our study reveals that reproduction can have nuanced trade‐offs with dominance and suggests that the study of dominance may benefit from explicitly considering variation in interaction rates as an additional factor affecting individuals.

## Graphical abstract

Females often have to trade‐off investment in reproduction versus social status. This study shows that after breeding, females lose more dominance interactions against other females, but that they also receive fewer aggressions from dominant males. The results suggest that breeding causes a change in the status of females, whereby they may need to invest less in same‐sex interactions because of increased access to resources through dominant males.

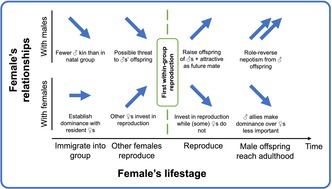

## 1 Introduction

In many group‐living species individuals occupy particular social positions, defined by their associations as well as agonistic and affiliative relationships with group members (Sosa 2016; Ward and Webster 2016). Dominance relationships, for example, are a prominent feature of many animal societies (Shizuka and McDonald 2015), with individuals' relative dominance dictating access to resources, such as reproductive opportunities (Ellis 1995). However, investment in reproduction must also be traded off with investments elsewhere (Williams 1966; Harshman and Zera 2007; Speakman 2008), such as maintaining body condition or engaging in social interactions (Cain and Ketterson 2013). Given that engaging in, and escalating, agonistic interactions is costly (Neat, Taylor, and Huntingford 1998; Briffa and Elwood 2004), individuals investing heavily in reproduction may become less likely to win agonistic interactions with group members.

While reproduction may detrimentally influence outcomes of individuals' intrasexual dominance interactions, reproducing may, conversely, positively alter individuals' interactions with the opposite sex. Reproducing individuals likely care for the offspring of at least one opposite‐sex group member (or more, if there is high within‐group relatedness among the opposite sex or if the reproducing individual has mated multiply). Such opposite‐sex group members should benefit from the survival of related juveniles (Hamilton 1964), and thus also from the survival of their carer. Reproducing individuals should therefore become more valuable to some members of the opposite sex, which may thus alter their interactions—for example, being more tolerant, or engaging in more affiliative or cooperative interactions—with such reproducing individuals. For example, in barbary macaques *Macaca sylvanus* breeding females receive more male grooming relative to non‐breeding females, despite these breeding females not increasing the grooming they give (Konečná et al. 2023). Accordingly, reproducing may modulate both intrasexual and intersexual social interactions of both reproducing and non‐reproducing individuals.

One situation where the influence of reproduction on intrasexual and intersexual interactions can be studied is sex‐stratified hierarchies (Kappeler et al. 2022), when primarily the subordinate sex invests in reproduction and intrasexual dominance is determined by individuals' relative competitive abilities. However, changes in *inter*sexual agonistic interactions should be driven by the costs and benefits to dominant‐sex individuals of aggressing particular subordinate, opposite‐sex group members—which may change with such subordinate‐sex group members' reproductive status, as outlined above. While complete dominance sex‐stratification may be a simplification (Kappeler et al. 2022), in most mammals, the dominant sex either disperses [as in many primates, e.g. chacma baboons *Papio ursinus* (Kappeler et al. 2022) and crested macaques *Macaca nigra* (Marty et al. 2016)] or invests more heavily in reproduction (as in spotted hyenas; Vullioud et al. 2019). In species where the dominant sex disperses, subordinate individuals are philopatric and thus likely have within‐group relatives and inherit their parents' social relationships (Ilany and Akçay 2016), as in rhesus macaques *Macaca mulatta* (de Waal 1996; Berman, Rasmussen, and Suomi 1997). Meanwhile, in species where the dominant sex invests in reproduction, studying reproduction‐related changes in tolerance by opposite‐sex individuals is largely redundant. Accordingly, it may be difficult to simultaneously investigate the effects of reproduction on intrasexual and intersexual agonistic interactions in most mammals. In contrast, in birds females often (i) are the dispersing sex (Greenwood 1980); (ii) are subordinate to males (as in many mammals) given their smaller body size; and (iii) invest heavily in reproduction (Romano et al. 2022). These features of many avian social systems raises the question of how reproductive investment and dominance may play out.

Here we examine the temporal dynamics of breeding and non‐breeding females' intrasexual and intersexual dominance interactions surrounding breeding seasons in vulturine guineafowl *Acryllium vulturinum* . This is a species in which females, the subordinate sex (Papageorgiou and Farine 2020) both invests highly in reproduction (Nyaguthii et al. 2025) and disperses (Klarevas‐Irby, Wikelski, and Farine 2021), whereas males (the dominant sex) is philopatric. This means that the body condition of individuals of the subordinate sex can be expected to change over time as a consequence of reproductive investment in egg production and incubation (Nyaguthii et al. 2025). For example, female ring‐necked pheasants *Phasianus colchicus* can lose 19% of their body condition from their investment in reproduction (Breitenbach and Meyer 1959).

The extreme bias in dispersal also means that females are unlikely to have any within‐group relationships prior to integrating into a new group post‐dispersal. Accordingly, changes in females' intrasexual dominance interactions surrounding breeding may be initially driven by their relative competitive abilities. Alternatively, such changes may be driven by females' temporary absenteeism. Breeding female vulturine guineafowl leave the social group for approximately 1 month while laying and incubating (Nyaguthii et al. 2025), and may thus be the primary targets of agonistic interactions upon their return to the group—overwhelming the returning females' ability to win interactions (Holekamp et al. 1993; van der Marel et al. 2023). By contrast, as adult males are socially dominant to females at all times (Papageorgiou and Farine 2020), changes in the rates of intersexual aggression received by females are unlikely to be driven by females' competitive abilities relative to males. Instead, changes in males' behaviour are more likely to be driven by either females' temporary absence or females' reproductive status (i.e., having offspring to whom they are related).

We specifically investigated outcomes of dominance interactions among females, and the rates of male aggression towards individual females, both before and after breeding in relation to females' reproductive status. This enabled us to quantify short‐term effects of reproduction in the subordinate, dispersing sex that occurred via both female–female interactions and male–female aggressions. We predict that breeding females should be less likely to win female–female dominance interactions against non‐breeding females after, but not before, breeding. This could arise because absentees typically lose more interactions after returning than prior to leaving (Holekamp et al. 1993; van der Marel et al. 2023). Alternatively, investment in reproduction may reduce breeding females' ability to win interactions. In vulturine guineafowl males are philopatric (Klarevas‐Irby, Wikelski, and Farine 2021), and thus likely related to one another's offspring. We therefore predicted that if changes in the level of intersexual aggression were driven by females' reproductive status, breeding females would face less male aggression than non‐breeding females after, but not before, breeding. In contrast, as returning absentees typically face enhanced aggression in diverse species (Holekamp et al. 1993; van der Marel et al. 2023), if changes in intersexual interactions were driven by breeding females' absenteeism, then breeding females should experience more male aggression than non‐breeding females after, but not before, breeding.

## 2 Methods

### 2.1 Data Collection

We studied a habituated vulturine guineafowl social group at Mpala Research Centre, Kenya, in a population that has been studied since 2016. The site comprises a mix of Savannah and dense, dry woodland habitat, with glades (open, grassy areas) spread throughout the study site. Seasonality at the study site is typically characterised by one short (October–November) and one long (March–May) rainy season each year. Breeding only takes place during extended wet periods (Papageorgiou et al. 2021; Nyaguthii et al. 2025) which can be up to twice a year but can also be skipped for several years. Between wet periods, the habitat is relatively dry (Rubenstein 2011; 2016), and no breeding behaviours take place. We collected breeding and interaction data between 4 March 2020 and 23 September 2022 as part of a long‐term research project, with the central analyses focusing on two breeding seasons, occurring in May–June 2020 and November–December 2020 (Figure S1) of one social group (no breeding took place between December 2020 and the end of 2022 due to an extended drought). Our data collection is limited to this group, and this period, because these birds live (and reproduce) predominately within a fenced compound, enabling us to follow them on foot and observe their interactions closely.

### 2.2 Ethical Note

Field methods were ethically reviewed and approved by the Max Planck Society Ethikrat Committee (2016_13/1) and the Animal Ethics Committee of the Australian National University (A2023/20 Farine), with data collection approved by the University of Exeter Biosciences Ethics Committee (eCORN002746 7.1). Data were collected under permits from Kenya Wildlife Service (KWS‐0016‐01‐21), the National Commission for Science, Technology and Innovation of Kenya (NACOSTI/P/16/3706/6465), the Wildlife Research and Training Institute (WRTI‐0286‐03‐23), and the National Environment Management Authority (NEMA/AGR/68/2017).

### 2.3 Study Species

Vulturine guineafowl live in stable social groups that range in size from 13 to 65 adult individuals (Papageorgiou et al. 2019) in dry woodland habitats in East Africa. Groups comprise more males than females and are characterised by steep dominance hierarchies. All adult males dominant to females (Papageorgiou and Farine 2020) and dominant individuals exclude subordinates from resources, resulting in reduced food intake rates of the latter (Papageorgiou and Farine 2020). Males' social influence over females' resource access is thus likely to be both considerable and mediated by agonistic interactions. Dispersal is strongly female‐biased, with males predominantly philopatric (Klarevas‐Irby, Wikelski, and Farine 2021). Field observations since 2016 suggest that social dominance among adult females may be unstable, and is thus unlikely to be regulated by conventions such as queueing (East and Hofer 2001; Field, Cronin, and Bridge 2006; Foerster et al. 2016) or social learning (Holekamp and Smale 1991) but rather, at least in part, influenced by individuals' physical size or condition. Social groups spend early mornings and late afternoons foraging and interacting on glades—typically feeding on grass leaves, roots, bulbs and seeds, as well as berries and small invertebrates (del Hoyo et al. 1992; Papageorgiou et al. 2021). Such prolonged exposure in open areas makes it easy to observe aggressive and submissive behaviours among group members (Papageorgiou and Farine 2020; Dehnen, Papageorgiou, et al. 2022).

Breeding can occur up to twice a year during wet periods, and starts with male–female pairs forming and moving separately from the remaining social group, with the male typically mate‐guarding the female against other males (Papageorgiou et al. 2019; Nyaguthii et al. 2025). While a number of females may form temporary pairs, only a portion of adult female group members typically attempt to breed in any given season (Nyaguthii et al. 2025). Females that breed lay eggs in a well‐hidden scrape on the ground on consecutive days, until they reach a clutch size of up to 15 eggs, although clutch sizes of 8–10 eggs are more typical in our population (Nyaguthii et al. 2025). The female then incubates the nest for approximately 23 days (del Hoyo et al. 1992). We have no observations of females receiving help during incubation (females of the closely related helmeted guineafowl *Numida meleagris* incubate alone; Elbin, Crowe, and Graves 1986), nor do females take any extensive foraging breaks during the incubation period. Meanwhile, males paired with incubating females re‐join the social group and often re‐pair with another female (Nyaguthii et al. 2025). High rates of nest predation in this ground‐nesting species mean that only some females that attempt to breed reach incubation and hatching (Nyaguthii et al. 2025). Within days of hatching, females return with their broods to the main group (Nyaguthii et al. 2025). Distinct subsets of male group members then help females raise their offspring, largely doing so by calling chicks to food items and covering chicks under their wings (Nyaguthii et al. 2025). Once the group has reformed, dominance interactions among group members can readily be observed.

### 2.4 Data Collection: Breeding

During breeding seasons, we monitored the breeding attempts of adult females in the social group. We endeavoured to follow paired females to determine the location of nests. We then monitored located nests to determine the number of eggs therein and the breeding stage reached. For the purposes of this study, we defined individuals that successfully hatched chicks as “breeders,” given that these individuals produced a clutch of eggs and incubated these for approximately 23 days (del Hoyo et al. 1992). We defined “non‐breeders” as those individuals that did not attempt to breed or those whose nests failed prior to hatching, given that this could happen after only few eggs were laid and without incubation. Accordingly, in our dataset, two females whose nests were predated—the first during the laying stage (season one: May–June 2020) and the other after approximately 1 week of incubation (season two: November–December 2020)—were categorised as non‐breeders. While we acknowledge that this is a somewhat arbitrary categorisation along a continuum of investment in breeding, our categorisation does present a clear and obvious distinction. Additionally, while females whose nests failed did invest in breeding, such females both invested to a lesser degree and returned to the group after only a short absence—while females who bred successfully were absent for the entirety of incubation, over 3 weeks.

For each breeding female in each season, we identified the first and last date of observed breeding behaviour (laying, incubating, or hatching). However, some females were absent for periods equivalent in duration to incubation and subsequently returned to the group with chicks despite their nests never being found. We treated these individuals (three of seven cases) as breeders. Additionally, some females' nests were found after they had started laying or incubating (prior to laying, nests are essentially only scrapes in the ground). For each female, we therefore subtracted 1 month (23 days incubation + 8 eggs laid on consecutive days) from the hatch date to estimate the start of their breeding behaviour. If this estimate was prior to the female's first known breeding behaviour, the estimate was used as the start of breeding instead. We then defined the “breeding period” for the social group in that season as the period of time between the first and last date of observed breeding behaviour from individuals defined as breeders in that group and season. Note that we do not consider post‐hatching parental care behaviour as breeding behaviour. This is because the precocial vulturine guineafowl chicks are relatively independent from a young age, and females return to the social group almost immediately upon chicks hatching—after which a considerable portion of offspring care is provided by male group members (Nyaguthii et al. 2023). Therefore, our among‐female interaction analysis considers dominance interactions both before breeding females leave the social group and once they return.

To approximate female investment in egg production, we weighed eggs from three females' clutches during season two using Kenex Eternity 100 scales. We then combined this egg weight data with female body weight measurements from trapping data. Specifically, we estimated female investment in egg production as a percentage of body mass via: 100 × no. eggs (8 or 10) × median egg weight/median female body weight. While this is unlikely to reflect all breeders' investments due to differences in clutch size, egg and body weights, it nonetheless provides an indication of female investment in egg production.

### 2.5 Data Collection: Dominance Interactions

We used all‐occurrence sampling (Altmann 1974) to record dyadic dominance interactions (both aggressive and submissive) among group members, typically during mornings and late afternoons when birds are most active, and also recorded the group composition and observation duration for each session. As the main social unit of vulturine guineafowl can split into temporary subgroups (Dehnen, Papageorgiou, et al. 2022), a new observation session was started when the group composition changed. Using this approach, we have established a large interactions dataset comprising different types of dominance interactions across years and social groups (Papageorgiou and Farine 2020; Dehnen, Papageorgiou, et al. 2022). We considered the actors of aggressive interactions and the recipients of submissive interactions to be the “winners”, and the recipients of aggressive interactions and the actors of submissive interactions to be the “losers”. We used subsets of this long‐term interaction dataset in all analyses. We removed all interactions involving individuals hatched during that breeding season.

### 2.6 Data Processing: Female–Female Interactions Surrounding the Breeding Season

We used data for each season in the among‐female interaction analysis, as follows. First, we created a 22‐week subset that was 11 weeks before and 11 weeks after the halfway point of the “breeding period” (defined above). We then removed any interactions from this subset that occurred during the breeding period and/or involved males, splitting the season's data into two approximately 8‐week subsets of female–female dominance interaction data: one before and one after breeding took place (see Figure S1). We then merged all data subsets into one dataset containing the data of all pre‐ and post‐breeding periods of both seasons. We thus obtained a winner–loser dataset containing interactions among and within breeding and non‐breeding females from both before breeding and after breeding for both seasons (see Figure S1 for timeline). Note that most individuals were present in both seasons (Table 1).

**TABLE 1:** Summary of female–female dominance data from each season and period therein, used in the among‐female interaction analysis.

Season/period	No. interactions	No. aggressive	No. submissive	No. dyads	No. breeders	No. non‐breeders
Season one/before breeding	173	108	65	59	3	14
Season one/after breeding	554	363	191	72	3	11
Season two/before breeding	327	239	88	79	4	11
Season two/after breeding	271	129	142	46	4	8
Overall	1325	839	486	116	5	17

### 2.7 Data Processing: Male–Female Aggressions Surrounding the Breeding Season

We used interaction data from the same time periods as the among‐female interaction analysis, above. We aggregated male‐aggression data for each female, such that we considered the total amount of male aggression a female received. While this loses some of the nuance in terms of differences in who gives the aggression, it was necessary to enable sufficient coverage across females. Specifically, for each data collection session, we calculated the number of male aggressions experienced by each female, as well as the number of males present and the session duration. The latter two measures were multiplied to produce a male‐hours variable capturing the exposure of females to males during each data collection session (and thus opportunity for females present to be aggressed by males). We then summed both the number of male aggressions and the male‐hours from all the data collection sessions for each female in each period in each season. This produced a total number of male aggressions received and total male‐hours for each female in each period, that is, before and after breeding, in each season. Each female was therefore present in the dataset at least twice (one value before and one after breeding), or four times if females were present in both seasons.

### 2.8 Analytical Approaches

Active dominance dynamics reflect reversals in existing dominance relationships (Dehnen, Arbon, et al. 2022) which, alongside passive demographic processes, can alter the position of individuals in inferred dominance hierarchies. Studying such dominance dynamics using longitudinal or repeated static hierarchies can be difficult. The interdependence among individuals (Sánchez‐Tójar, Schroeder, and Farine 2018) mean that errors in estimating hierarchy positions are highly correlated. For example, if an individual is ranked incorrectly then, by definition, other group members will also be ranked incorrectly. Additionally, measurement uncertainty is often not explicitly considered and the time steps at which hierarchies are updated can influence hierarchy dynamics (Strauss and Shizuka 2022). Furthermore, passive demographic processes can drive hierarchy dynamics (Strauss 2023), which we were not interested in here. Accordingly, rather than generating hierarchy positions, our analyses focused on interaction‐level data and used generalised linear mixed effects models to investigate changes in the patterns of interactions, as done in previous studies of interaction outcomes (Wilson, Grimmer, and Rosenthal 2013; Lane, Wilson, and Briffa 2020).

Throughout, we assessed model fits using the R package DHARMa (Hartig 2022), and assessed statistical significance of model fixed effects using type‐II (for models with no interaction terms) and type‐III (for models with interaction terms) Wald *χ* ^2^‐tests in the R package car (Fox and Weisberg 2019) unless specified otherwise. We conducted all statistical analyses using R version 4.1.2 (R Core Team 2021), aside from the ASReml models, which were run in R version 4.3.0, and considered *p* < 0.05 as significant. We used the R packages sjPlot (Lüdecke 2023), ggplot2 (Wickham 2016), igraph (Csardi and Nepusz 2006), and cowplot (Wilke 2020) to create figures.

#### 2.8.1 Dyadic‐Interaction Model Overview

In our interaction‐level datasets there was one winner and one loser for every interaction. As winner–loser data consist of two interacting individuals but only one outcome, we randomly assigned one individual as the “focal” (focal_ID) and one as the “interactor” (interactor_ID) for each interaction. In such dyadic contest data, individual “a” winning against individual “b” could be represented as either focal_a_ beats (1) interactor_b_, or focal_b_ loses (0) to interactor_a_. The outcome (1/0) is therefore entirely dependent on the allocation of the two individuals to focal and interactor roles, which is important in datasets comprising multiple observations per individual (Wilson et al. 2011), as is the case for our interaction data. In our analyses using interaction‐level data, we therefore associated each observation (interaction) with two levels (focal_ID and interactor_ID) of a single, additive random effect to model focal and interactor identities and thereby control for pseudoreplication.

#### 2.8.2 Among‐Female Interaction Analysis

To assess whether the probability of a female winning intrasexual dominance interactions changed as a consequence of breeding, we fitted a generalised linear mixed‐effects model to the dataset of female–female interactions surrounding the breeding season using the glmer function of the R package lmerMultiMember (van Paridon, Bolker, and Alday 2023). This package provides a wrapper for the glmer/lmer functions of the R package lme4 (Bates et al. 2015), and allows the modelling of focal and interactor identities as a single, additive random effect for winner–loser data with multiple observations per individual. This replicates the random effects structure of ASReml models in similar analyses (Wilson et al. 2011; Lane, Wilson, and Briffa 2020). We chose to use lmerMultiMember over ASReml software as lmerMultiMember allows for binomial error structures (which are appropriate for our data, while ASReml allows only Gaussian). As the lmerMultiMember package is relatively new, we replicated the among‐female interaction analysis (see below) using the R package ASReml, which has been widely used for modelling dyadic interactions for over a decade (Wilson et al. 2011). Our ASReml replicate confirms that model outputs are qualitatively the same regardless of the R package used (see Data S1).

We fitted a binomial variable *focal_won*, capturing whether the focal won (1) or lost (0) the dominance interaction, as the response variable and used a binomial error structure. Fixed effects included: period, whether the interaction took place before or after breeding; dyadic breeding contrast, a three‐way categorisation of the focal and interactor combined breeding status (either focal = breeder & interactor = non‐breeder, focal = non‐breeder & interactor = breeder, or focal & interactor were of the same breeding status). Under the hypothesis that breeders are less likely to win against non‐breeders—and *vice versa* for non‐breeders against breeders—post‐breeding, the interaction term between period and dyadic breeding contrast was predicted to be significant. Specifically, predicted values were predicted to diverge for breeding and non‐breeding individuals after breeding, such that non‐breeding individuals were more likely to win against breeding individuals. We also included season, that is, which season the interaction took place in (season one or two), as a fixed effect (due to having only two levels). Random effects included: focal_ID and interactor_ID (defined above) as a single, additive random effect as outlined above; and Dyad_ID, an ID unique to the interacting dyad (irrespective of focal and interactor allocation) as a further random effect. We tested the significance of random effects using likelihood ratio tests comparing models with and without the random effect in question.

#### 2.8.3 Male–Female Interaction Analysis

To assess whether the level of male aggression experienced by females varied according to breeding status and period, we fitted a generalised linear mixed‐effects model to the dataset on male–female aggressions surrounding the breeding season. We fitted the number of male aggressions received (*num_aggro*) as the response variable. To account for opportunity for females to be aggressed by males, we log‐transformed the *num_male_hours* variable (which captures females' exposure to males) to produce the variable *log_num_males_hours* and fitted this as an offset. We fitted both period and the female's breeding status (*breeder_status*), as well as the interaction term between them, as fixed effects. As in other models, we wanted to control for season, which we fitted as a fixed effect due to having only two levels. We fitted the female's identity (*ID.original*) as a random effect, given that each female contributed at least two datapoints. We fitted the initial model with a Poisson error distribution using the R package lme4 (Bates et al. 2015), but this suffered from overdispersion. We thus fitted a negative binomial model instead using the R package glmmTMB (Brooks et al. 2017). This model converged without problems and was used for statistical analysis.

## 3 Results

### 3.1 Egg Investment

Vulturine guineafowl eggs (*n*_eggs_ = 19; *n*_females_ = 3) weighed a median of 46 g (range: 43–52 g), with the median body weight of a female (*n*_weights_ = 882; *n*_females_ = 533) being 1380 g (range: 1030–1840 g). Given these estimates of egg and female body weights, and clutch sizes of 8–10 eggs (Nyaguthii et al. 2025), egg production represented approximately 27%–33% of females' body weight and thus a substantial investment to females alongside incubation effort.

### 3.2 Among‐Female Interaction Analysis

**FIGURE 1:**
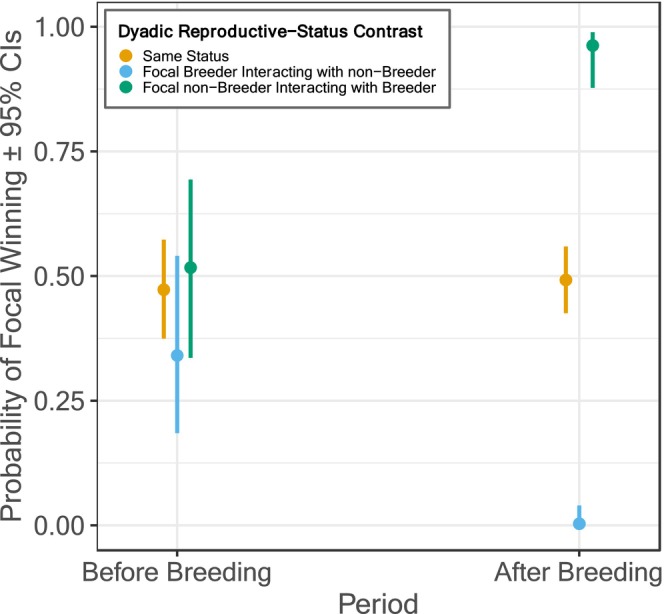
Breeding females were more likely to lose interactions with non‐breeding females after, but not before, breeding. Dyads where the focal and interacting individual are of the same breeding status (yellow points) comprise both breeder–breeder and non‐breeder–non‐breeder interactions. Results are based on 1325 interactions among five breeding females and 17 non‐breeding females.

Our among‐female interaction analysis involved 1325 female–female interactions from a total of 116 dyads. These consisted of 5 females that bred and 17 females that did not (see Table 1 for season‐ and period‐level details), with two females breeding in both seasons and three breeding in only one season (Figure S1). We found no evidence for either period (*χ* ^2^_1_ = 0.142, *p* = 0.706) or dyadic breeding contrast (*χ* ^2^_2_ = 1.848, *p* = 0.397) to affect focal winning probability in our among‐female interaction analysis. Likewise, we found no significant difference between focal individuals' winning probabilities between seasons (*χ* ^2^_1_ = 0.0525, *p* = 0.819). However, as predicted, there was a significant interaction between period and dyadic breeding contrast (*χ* ^2^_2_ = 33.309, *p* < 0.001). Specifically, non‐breeders experienced an increased probability of winning against breeders after the breeding period, while breeders experienced a corresponding decreased probability of winning against non‐breeders after the breeding period. Meanwhile, breeders and non‐breeders appeared to be equally likely to win before breeding (Figure 1).

Testing of random effects in the among‐female interaction analysis revealed between‐individual differences in competitive ability. Specifically, the model including the additive term of focal ID and interactor ID outperformed the model excluding this term (*χ* ^2^ = 751.524, *p* < 0.001), indicating that individuals differed in competitive ability beyond their breeding status. Adding dyad ID as a random effect did not further improve the model beyond the additive effect of the interacting individuals' identities (*χ* ^2^ = 0.082, *p* = 0.774) (Table 2). This result suggests that interactions among females in the among‐female interaction analysis dataset are highly transitive.

**TABLE 2:** Likelihood ratio tests comparing among‐female interaction analysis models that differ in their random effects structure.

Model random effect structure	*AIC*	*Log‐likelihood*	*χ* ^*2*^	*p*
Season	1779.34	−882.67		
**Season + additive individual IDs**	**1029.82**	**−506.91**	**751.52**	**< 0.001**
Season + additive individual IDs + dyad ID	1031.74	−506.87	839	0.774

*Note:* Each model is a contrast to the model above, with models in which the additional random effect significantly improved model performance highlighted in bold.

### 3.3 Male–Female Interaction Analysis

**FIGURE 2:**
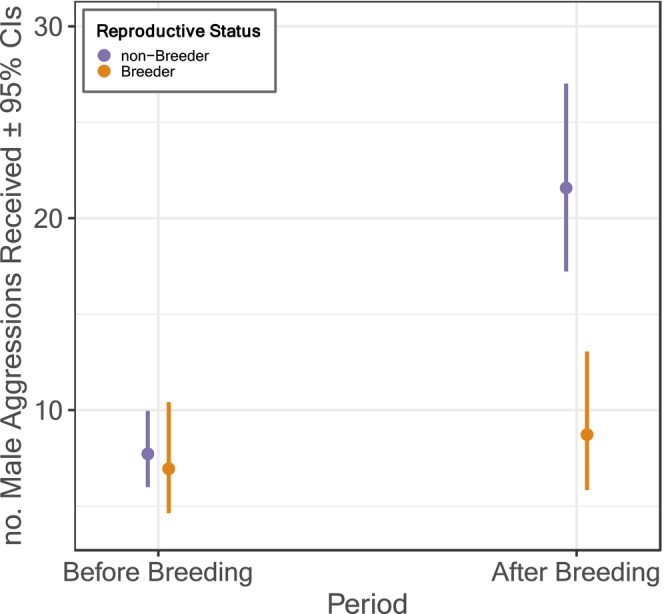
Breeding females received less aggression from males than non‐breeders after, but not before, breeding. The number of aggressions received by females was corrected to account for females' exposure to males, calculated as log(total number of male‐hours a female was exposed to), using an offset term in the model. Results are based on intersexual agonistic interactions directed by males towards five breeding females and 17 non‐breeding females.

We found that the number of male aggressions females faced differed between seasons (being higher in season two, *χ* ^2^_1_ = 56.780, *p* < 0.001). We found no evidence for a difference in aggression faced between breeding and non‐breeding females across all periods and seasons (*χ* ^2^_1_ = 0.270, *p* = 0.604). However, while the number of male aggressions faced by females was the same for breeders and non‐breeders before breeding, the level of aggression faced diverged after breeding according to females' breeding status. Specifically, non‐breeding females faced considerably more male aggression than breeding females after breeding (Figure 2), as highlighted by the significant interaction between period and breeder status (*χ* ^2^_1_ = 10.709, *p* = 0.001). This enhanced aggression faced by non‐breeding females after the breeding period likely largely drove the greater aggression faced by females overall after breeding relative to before breeding (*χ* ^2^_1_ = 80.110, *p* < 0.001). However, the difference in aggression received before versus after breeding could also be a product of sampling method (see discussion).

## 4 Discussion

In many group‐living species reproductive success is skewed towards dominant individuals (Ellis 1995; Clutton‐Brock and Huchard 2013a). These breed more often, produce larger offspring, or produce more offspring per reproductive event (Clutton‐Brock and Huchard 2013b). However, reproduction can also be a predictor of future dominance outcomes. Our analysis of female–female dominance interactions surrounding breeding seasons revealed that, while breeders had an equal probability of winning dominance interactions with non‐breeders before breeding, this reduced considerably after breeding (Figure 1) and remained so for at least 3 months (see Data S1). We also found no evidence that breeders' winning probability declined further or recovered between two post‐breeding periods (Figure S2, Table S1). It is difficult to determine whether this suggests that our results were not driven by individuals in decline choosing to breed (akin to terminal investment; Clutton‐Brock 1984), given the limited parameter space for breeders to decline further. However, the two breeding seasons were in relatively close succession (approximately 6 months apart) and we detected no pre‐breeding differences in breeding and non‐breeding females' winning probabilities, suggesting that at least females breeding in the first season were not in decline. Examination of random effects in our among‐female interaction analysis further suggested that dominance in female vulturine guineafowl was highly transitive, and that females differed in competitive ability beyond their breeding status alone (Table 2). Meanwhile, our analysis of male–female interactions surrounding breeding seasons showed that breeding females were the targets of less intersexual, male aggressive interactions when compared to non‐breeding females after, but not before, breeding. These patterns are relatively strong overall, but we acknowledge that our data were limited to five breeding and 17 non‐breeding females across two seasons (due to drought limiting opportunities for breeding). The generalisability of our results therefore warrants scrutiny; these are initial findings on temporal dynamics in female dominance interactions in relation to breeding warrange further exploration both using larger datasets and in other species.

### 4.1 Female Reproduction and Short‐Term Intrasexual Interaction Outcomes

Females' intrasexual interaction outcomes could have been driven by multiple, non‐exclusive mechanisms. Breeding female vulturine guineafowl invest considerably in reproduction by producing a large clutch of eggs (constituting ~30% of their body mass) that they incubate alone (Nyaguthii et al. 2025). Further, breeding comes on the back of dry seasons during which birds are food limited (Papageorgiou et al. 2021). Meanwhile, non‐breeding females may continue to forage during the resource‐rich wet season. We therefore hypothesise that reduced condition arising from breeding drives the reduction in the probability of breeding females winning dominance interactions. Our analysis of interactions surrounding trapping events outside of the breeding season however suggests that, while structural size has some influence on interacting outcomes, body condition appears to play a lesser role (Figure S6). Nevertheless, at extreme differences in body condition the individual in vastly superior condition always won (see Figure S6). If reproductive investment results in such extreme differences in body condition among breeders and non‐breeders, condition could indeed be the proximate mechanism driving the observed divergence in breeders' and non‐breeders' winning probabilities after breeding.

A non‐mutually exclusive, alternative possible driver of breeders' post‐breeding decline in winning probability is caused by the absence of breeding females. Breeding females are effectively removed from the social group for at least the duration of incubation, which lasts around 1 month (see horizontal lines in Figure S1). In many species, recent dyadic interaction outcomes determine individuals' behaviour in future interactions within that dyad (Dehnen, Arbon, et al. 2022). The absence of breeding females from the social group may thus “release the shackles” on prior subordinates—even in the absence of changes to intrinsic attributes such as body condition. In spotted hyenas, for example, females returning to a social group after being absent for over 6 months received severe aggression and suffered reduced dominance relative to their hierarchy positions prior to leaving (Holekamp et al. 1993). Similarly, experimental removal and reintroductions of individual monk parakeets *Myiopsitta monachus* highlight that temporary, 8 days absences can cause returning individuals to receive enhanced aggression and consequently drop down the hierarchy (van der Marel et al. 2023). Accordingly, elucidating the mechanism driving the relationship between breeding status and interaction outcomes in female vulturine guineafowl will require experimental manipulation, for example, through supplementary feeding or experimental removals, or the fine‐scale tracking of individuals' condition.

How and why reproduction influences interaction outcomes likely depends on how reproduction alters the determinants of dominance (Dehnen, Arbon, et al. 2022) among breeding and non‐breeding females. These are likely to be largely dictated by the reproductive biology and the context of competitive interactions of the species. For example, breeders should be more likely to win agonistic interactions in species where (a) individuals frequently compete for monopolisable resources that represent tangible fitness benefits to breeders and (b) physical reproductive investment is relatively low. In female parasitoid wasps *Goniozus nephantidis* and *Eupelmus vuilleti*, contests for access to hosts within which to lay their eggs are won by the individual that is carrying more eggs (Stokkebo and Hardy 2000; Mohamad, Monge, and Goubault 2010). Similarly, reproductively experienced carrion roller beetles *Canthon cyanellus cyanellus* are more likely to win contests for food balls than naïve individuals (Chamorro‐Florescano, Favila, and Macías‐Ordóñez 2017). As the respective authors suggest, these effects are likely driven by individuals' asymmetric resource valuation, which arises from the difference in reproductive benefits of winning among breeding and non‐breeding individuals. Conversely, breeders should be less likely to win agonistic interactions post‐breeding in species where (i) individuals rarely compete for monopolisable resources (or in which a single monopolisable resource represents a smaller fitness payoff), (ii) breeders suffer reduced physical condition (i.e., intrinsic attributes; Dehnen, Arbon, et al. 2022) as a consequence of short and intense reproductive investment (as is likely the case in many birds), and/or (iii) individuals are temporarily absent from typically stable social groups (Holekamp et al. 1993; van der Marel et al. 2023) while reproducing. These all warrant substantially more study, especially in species where breeders are the subordinate sex.

### 4.2 Female Reproduction and Short‐Term Intersexual, Male–Female Aggressions

We found lower levels of intersexual, male aggression experienced by breeding females, relative to non‐breeding females, after but not before breeding. This finding suggests that changes in intersexual agonistic interactions were not driven by females' temporary absence from the group, but by females' reproductive status per se. Several processes could have driven this finding. Vulturine guineafowl societies are characterised by female dispersal and male philopatry (Klarevas‐Irby, Wikelski, and Farine 2021), which likely lead to relatively high levels of relatedness among males of the same social group. Males are thus likely related to the offspring of one or more breeding females, which is reflected in their caring for juveniles that are unlikely to be their own offspring (Nyaguthii et al. 2025). Being less aggressive, that is, tolerant, towards mothers of such related offspring could thus carry indirect fitness benefits for males. In other words, successful reproduction elevates the female's social position among male group members.

The observed pattern could also be driven by males directing more aggression towards non‐breeding females. Due to female‐biased dispersal (Klarevas‐Irby, Wikelski, and Farine 2021), females that have dispersed and do not breed are unlikely to be closely related to any chicks produced by females in the group and thus incentivised to harm them (Ellis et al. 2022). Indeed, juvenile‐directed female aggression is common among other species characterised by cooperative breeding (Koenig and Dickinson 2016) or dominance hierarchies (Lukas and Huchard 2019), likely driven by female–female competition. Vulturine guineafowl males help a particular female raise her offspring, providing care that includes protecting chicks from other adult group members (Nyaguthii et al. 2025). The observed difference in aggression received by breeding and non‐breeding females after breeding may therefore be driven by males protecting chicks by expressing enhanced aggression towards such non‐breeding females. This latter explanation is in line with the model‐predicted values: after breeding the level of male aggression received increased for non‐breeding females but remained relatively stable for breeding females (Figure 2). However, we advise some caution when interpreting variation in predicted values *between* periods, that is, before versus after breeding, as variation therein may be an artefact of the sampling method. Specifically, data were collected via all‐occurrence sampling, for which observed dyadic rates likely decrease with increasing group size. As sampled subgroups are typically larger pre‐breeding (when the social group is cohesive) than post‐breeding (when broods and associated helpers move independently; Nyaguthii et al. 2025), it is unclear whether the difference in pre‐ versus post‐breeding predicted values reflect true rates or an artefact of temporary changes in social structure. Accordingly, determining the mechanism driving breeding‐status related differences in levels of male aggression received by breeding and non‐breeding females after breeding is not possible with the present dataset. However, within‐period differences—between breeders and non‐breeders before breeding or after breeding—should not be affected by any such effects.

The short‐term changes in females' intrasexual and intersexual agonistic interactions may translate into tangible food‐intake outcomes. Dominant males aggressively exclude subordinate individuals from monopolisable food patches, reducing their food intake rates (Papageorgiou and Farine 2020). Biased male aggression may thus result in breeding females gaining greater access to food resources, ahead of non‐breeding females (especially after breeding). However, breeding females also rarely win against non‐breeding females after the breeding period. Accordingly, breeding females may be less able to access food than non‐breeding females in the absence of males. Herein lies on the important insights from our study—that post‐breeding social interactions are likely to be shaped by how breeding alters the patterns of relatedness within social groups.

### 4.3 Long‐Term Consequences of Reproduction

Kinship dynamics occur when individuals' relatedness to their social group changes over time as a consequence of demographic processes and often operate in group‐living species with pluralistic breeding (Croft et al. 2021; Ellis et al. 2022). For example, in chimpanzees *Pan troglodytes* , dispersal is strongly female‐biased (Newton‐Fisher 2014) meaning that, while initially low immediately after immigrating, females' relatedness to group members increases over time via philopatry of male offspring (Ellis et al. 2022). As dispersal in vulturine guineafowl is likewise female‐biased (Klarevas‐Irby, Wikelski, and Farine 2021), as in most birds (Greenwood 1980), similar kinship dynamics likely operate in vulturine guineafowl societies. Vulturine guineafowl groups also feature pluralistic breeding (Nyaguthii et al. 2025), which may be key to such kinship dynamics (Ellis et al. 2022). Relatedness of female vulturine guineafowl to their group members can thus be expected to increase over time as they reproduce. Given that individual kinship dynamics drive the inclusive fitness payoffs associated with helping and harming behaviours (Croft et al. 2021), female vulturine guineafowl may, as a consequence of generating philopatric sons, experience greater helping (e.g., tolerance, helper offspring care), and reduced harming (e.g., aggression) from their group members over time. Indeed, preliminary data (limited to the adult sons of a single female) suggest that male vulturine guineafowl exhibit role‐reversed nepotism, whereby adult males are less aggressive towards their own mother than towards other female group members (Figure S5). Similar effects are found in spotted hyenas *Crocuta crocuta* , where cubs are tolerant of their (subordinate) fathers, towards which they direct less aggression than towards unrelated males (van Horn, Wahaj, and Holekamp 2004). Aside from kinship dynamics and associated benefits, females that breed successfully could also become more attractive to males, given that they have a track record of proven fecundity (Anderson 1986; Nichols et al. 2010). Our field observations suggest that forced copulations among vulturine guineafowl are uncommon (T. Dehnen, *personal observation*), meaning that females likely have considerable reproductive control. Males may thus exhibit enhanced tolerance towards previously breeding females that are potential mates. Reproduction may therefore have long‐term consequences for vulturine guineafowl females' social experience via multiple possible pathways. While examining long‐term patterns of social integration and interactions in wild animal groups is not without its difficulties, we encourage future studies to investigate long‐term transitions in individuals' social “positions” in relation to past breeding success.

**FIGURE 3:**
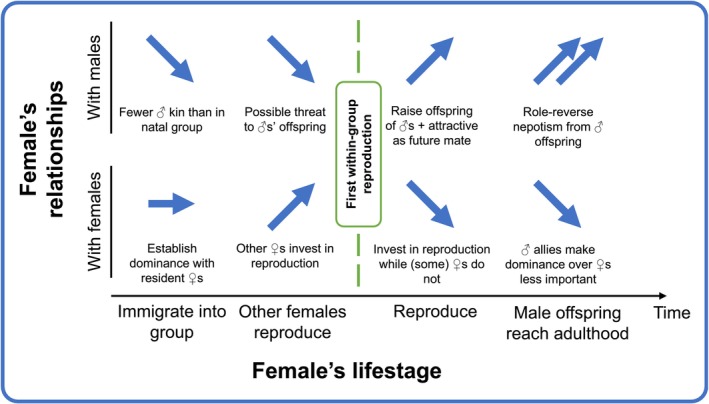
Schematic of females' possible relationship transitions with both male and female adult group members over time—from immigration through to different reproductive events and, eventually, acquiring male within‐group kin and associated benefits. Arrows represent the directionality of the changes in relationships that female group members have with males (affiliative) and females (agonistic) during each lifestage. Arrows pointing upwards indicate greater tolerance (from males) or increased status (among females). Arrows pointing downwards indicate reduced tolerance (from males) or reduced social status (among females).

By influencing females' relationships with males, breeding successfully could also have long‐term consequences for females' investment in intrasexual dominance. Attaining and maintaining social dominance is costly, as individuals have to invest time (Ebensperger and Hurtado 2005) and energy (Neat, Taylor, and Huntingford 1998; Briffa and Elwood 2004) in engaging in and escalating dominance interactions. However, females that have successfully bred may gain access to high‐value resources via an alternate route—tolerance from related and unrelated males (as discussed above). This could allow previously breeding females to reduce investment in intrasexual dominance, and simultaneously allocate more resources to future reproduction. Thus, in the long‐term, breeding successfully may allow females to break free of a potential condition‐ and dominance‐mediated negative feedback loop between resource access and reproduction. Meanwhile, females that lack such male tolerance can only invest in intrasexual dominance—female vulturine guineafowl never win a dominance interaction against a male—so that they may acquire the resources necessary to breed themselves and, ultimately, generate their own set of tolerant males. Females' social relationships with male and female group members may thus change over time after entering the social group as a consequence of kinship dynamics (see Figure 3). Future work should investigate to what degree females benefit from male tolerance resources (e.g., quantifying food consumption) and invest in intrasexual dominance (e.g., receiving and initiating agonistic interactions), as a function of current and prior breeding success as well as the presence of particular male “allies”. Such effects could be studied in other group‐living species with both male‐biased dominance and agonistic interactions among females, as is likely the case in many mammals and birds.

## 5 Conclusion

In vulturine guineafowl, agonistic interactions with conspecifics determine individuals' access to resources and correspondingly influence food intake rates (Papageorgiou and Farine 2020). Our data suggest that reproduction has contrasting consequences for vulturine guineafowl females' agonistic interactions with female and male group members. Female resource access may thus be context‐dependent. Our study highlights a dynamic relationship between reproduction and social dominance in group‐living species, and adds reproductive status as a further possible driver of dominance dynamics (Combes and Altmann 2001; Strauss and Holekamp 2019; Portugal et al. 2020). Given our limited dataset, we encourage future research to identify tangible long‐term effects of breeding‐related intersexual tolerance. For example, one could build on this study and previous work (Papageorgiou and Farine 2020) by relating individual food consumption rates to intrasexual dominance interactions, according to reproductive status and the presence of tolerant dominant‐sex group members. More broadly, our empirical findings highlight that interaction rates alongside interaction outcomes dictate how individuals experience their competitive social landscapes. A key insight is that reproduction is likely to alter the social position of females within their social group. With our study, we hope to stimulate more research concerning the patterns and dynamics of dominance interactions (not just their outcomes) and relationships both within the subordinate sex and between the sexes, areas that have historically been understudied but are increasingly recognised as important (Cain and Ketterson 2013; Clutton‐Brock and Huchard 2013a, 2013b; Krieg and Getty 2020; Davidian et al. 2022; Kappeler et al. 2022).

## Author Contributions

**Tobit Dehnen:** data curation (lead), formal analysis (equal), methodology (equal), writing – original draft (equal), writing – review and editing (equal). **Brendah Nyaguthii:** data curation (equal). **Wismer Cherono:** data curation (equal). **Neeltje J. Boogert:** formal analysis (equal), methodology (equal), supervision (equal), writing – original draft (equal), writing – review and editing (equal). **Damien R. Farine:** conceptualization (equal), formal analysis (equal), funding acquisition (equal), methodology (equal), supervision (equal), writing – original draft (equal), writing – review and editing (equal).

## Conflicts of Interest

The authors declare no conflicts of interest.

## Supporting information


Data S1

## Data Availability

Data and code for all empirical aspects of this work are available at https://doi.org/10.6084/m9.figshare.28139600.v1.
